# KPI5 Is Involved in the Regulation of the Expression of Antibacterial Peptide Genes and Hemolymph Melanization in the Silkworm, *Bombyx mori*

**DOI:** 10.3389/fimmu.2022.907427

**Published:** 2022-05-20

**Authors:** Jingya Heng, Huawei Liu, Jiahui Xu, Xuan Huang, Xiaotong Sun, Runze Yang, Qingyou Xia, Ping Zhao

**Affiliations:** ^1^ State Key Laboratory of Silkworm Genome Biology, Biological Science Research Center, Southwest University, Chongqing, China; ^2^ Chongqing Key Laboratory of Sericultural Science, Chongqing Engineering and Technology Research Center for Novel Silk Materials, Southwest University, Chongqing, China

**Keywords:** *Bombyx mori*, Kunitz-type protease inhibitor, innate immunity, antimicrobial peptide, hemolymph melanization

## Abstract

Kunitz-type protease inhibitors (KPIs) are ubiquitously found in many organisms, and participate in various physiological processes. However, their function in insects remains to be elucidated. In the present study, we characterized and functionally analyzed silkworm KPI5. Sequence analysis showed that KPI5 contains 85 amino acids with six conserved cysteine residues, and the P1 site is a phenylalanine residue. Inhibitory activity and stability analyses indicated that recombinant KPI5 protein significantly inhibited the activity of chymotrypsin and was highly tolerant to temperature and pH. The spatio-temporal expression profile analysis showed that KPI5 was synthesized in the fat body and secreted into the hemolymph. *In vivo* induction analysis showed that the expression of *KPI5* in the fat body was significantly upregulated by pathogen-associated molecular patterns (PAMPs). Binding assays suggested that KPI5 can bind to pathogens and PAMPs. *In vitro* pathogen growth inhibition assay and encapsulation analysis indicated that KPI5 can neither kill pathogenic bacteria directly nor promote the encapsulation of agarose beads by silkworm hemocytes. Recombinant protein injection test and CRISPR/Cas9-mediated knockdown showed that KPI5 promotes the expression of antimicrobial peptides (AMPs) in the fat body. Moreover, the survival rate of individuals in the *KPI5* knockdown group was significantly lower than that of the control group after pathogen infection. Phenoloxidase (PO) activity assays showed that KPI5 significantly inhibited the hemolymph PO activity and melanization induced by PAMPs. These findings suggested that KPI5 plays a dual regulatory role in innate immunity by promoting the expression of antimicrobial peptides in the fat body and inhibiting hemolymph melanization. Our study furthers the understanding of the function of insect KPIs and provides new insights into the regulatory mechanism of insect immune homeostasis.

## Introduction

As one of the most widely distributed groups of organisms on Earth, insects have evolved a complex and efficient defense system, of which innate immunity is an important defense line for insects to resist the invasion of pathogens (1, 2). The innate immune system of insects is comprised by humoral and cellular immunity (2). The insect immune response mainly includes the following steps: pathogen recognition and pathogen-associated molecular patterns (PAMPs) on the surface of pathogenic microorganisms are specifically recognized by pattern recognition receptors (PRRs) (3); then, non-self-recognition triggers a series of downstream signals, such as serine protease cascades, which activate the downstream innate immune system and produce immune effectors to eliminate pathogens (2). When pathogens break through physical barriers, such as the integument and enter the hemolymph, different types of hemocytes clear pathogens through cellular immune responses such as phagocytosis, encapsulation, and nodulation (4, 5). Insect humoral immunity exerts immune effects mainly through two reactions (6–8): the production of antibacterial peptides (AMPs) with bactericidal effects, and melanization, which activates prophenoloxidase (PPO) and catalyzes the production of melanin to remove pathogens.

The innate immune system enables insects to defend against the invasion of various pathogens; however, excessive activation of the immune system is also harmful to the insect. When the melanization reaction is over-activated, the excess production of reactive oxygen species and quinine causes damage to insect tissue function and can even lead to death (9). Insects with excessive immune responses sometimes have intestinal flora disorders, nerve degeneration, and shortened survival (10, 11). In addition, the immune response is an energy-consuming process that competes with other physiological processes for nutrients; thus excessive immune responses significantly affect overall physiological metabolism (12). Therefore, the intensity and duration of activation of insect immune responses must be tightly and precisely regulated.

Previous studies have shown that extracellular serine protease cascades (EPCs) in insect humoral immunity are regulated by protease inhibitors. EPCs rapidly amplify and transmit pathogen invasion signals downward, and the terminally activated serine protease cleaves PPO to phenoloxidase (PO), which activates the melanization reaction, or cleaves the cytokine-like protein Spätzle, which activates the Toll signaling pathway to induce the production of AMPs (13, 14). Immune regulators targeting insect EPCs include serine protease inhibitors (serpins) and Kunitz-type protease inhibitors (KPIs). Several serpins have been reported to be involved in the regulation of insect immune processes by inhibiting the activation of PPO and Toll pathways (15, 16). The tobacco hornworm, *Manduca sexta* serpin1J and serpin3-7 regulate humoral immune responses by covalently binding to serine proteases of EPCs (17–21). *Bombyx mori* serpin5 targets HP6 and SP21 to downregulate Toll and PPO pathways (22). Three serpins (SPN40, SPN55, and SPN48) have been found to inhibit melanization and the Toll pathway in the mealworm, *Tenebrio molitor* (23). Studies have shown that KPIs may regulate hemolymph melanization in *M. sexta* and *B. mori* (24, 25). However, it remains unclear whether insect KPIs are involved in the regulation of humoral immunity.

KPIs are ubiquitous protease inhibitors in organisms, usually with one or more Kunitz domains that form a stable spatial structure through three pairs of typical intrachain disulfide bonds (26, 27). Although KPIs are conserved in their secondary structures, their functions vary in different species. Previous studies have shown that KPIs are involved in coagulation, inflammatory regulation, cell proliferation, antimicrobial infection and other processes in invertebrates (28–30). In the castor bean tick, *Ixodes Ricinus*, CPI has a typical Kunitz domain that binds specifically to human plasma FXIa, FXIIA, and kallikrein to inhibit the coagulation pathway and slightly inhibit fibrinolysis *in vitro* (31). Simukunin, a single-domain KPI from the black fly, inhibits factor Xa, elastase, and cathepsin G, suggesting that it regulates both blood clotting and inflammatory responses in the host (32). In the Greater wax moth, *Galleria mellonella*, KTPI might be responsible for the protection of other tissues against proteolytic attack by trypsin-like protease(s) from the larval midgut during metamorphosis, and might play a role in the proliferation of cells in the fat body and integument (28). In *B. mori*, hemolymph KPI CI-b1 is a lipopolysaccharide-binding protein that is speculated to be a mediator which scavenges intruding bacteria (33). In silkworm cocoon, SPI51, the most abundant KPI, inhibits fungal growth by binding to *β*-d-glucan and mannan on the surface of fungal cells (30).

In 2012, Zhao et al. identified 80 serine protease inhibitors at the whole-genome level in silkworm, including serpin, Kunitz_BPTI, Kazal, TIL, amfpi, Bowman-Birk, Antistasin, WAP, Pacifastin, and alpha-macroglobulin (34). Ten KPIs were identified in silkworms. In our previous studies, the activity of the Kunitz-type chymotrypsin inhibitor CI-13 (KPI5) was detected in the hemolymph of 425 silkworm strains (unpublished data), suggesting that it may play an important role in silkworm hemolymph. In the present study, we explored the function of KPI5 and found that it promotes the expression of AMPs in the fat body, inhibits hemolymph melanization and plays an essential role in silkworm innate immunity. This study promotes the understanding of the function of insect KPIs and provides new insights into the regulatory mechanism of insect immune homeostasis.

## Materials and Methods

### Materials and Sample Collection

The Dazao strain of *B. mori* was reared on fresh mulberry leaves at room temperature with a photoperiod of 12 h light/12 h dark and 70% relative humidity. *Escherichia coli*, *Staphylococcus aureus*, *Micrococcus luteus*, and *Saccharomyces cerevisiae* were maintained in the State Key Laboratory of Silkworm Genome Biology at Southwest University of China. *Bacillus bombyseptieus*, a common bacterial pathogen that causes bacterial black thorax septicemia in silkworms, was kindly provided by Professor Yanwen Wang (Shandong Agriculture University, China). All bacterial strains were cultured in LB medium at 37°C for 12 h; *S. cerevisiae* was cultured in YPD medium at 28°C for 36 h. Various tissues from 3-day-old-fifth-instar larvae were collected for RNA isolation. Fat bodies, hemocytes and hemolymph from 3-day-old-fourth-instar larvae to 1-day-old pupae were collected for RNA isolation and protein extraction. Through silkworm gastropod puncture, the hemolymph was collected into a pre-cooled 1.5 mL microcentrifuge tube, which contained 400 μL phosphate buffer saline (PBS) and an appropriate amount of phenylthiourea (PTU). The hemocytes were collected by centrifugation at 800 × g for 5 min at 4°C. Other tissues were dissected with sterile scissors and forceps, then placed in sterile PBS for washing, and finally collected in a 1.5 mL microcentrifuge tube, and frozen at -80°C.

PAMPs (Sigma-Aldrich, St. Louis, MO, USA), including peptidoglycan (PGN), lipopolysaccharide (LPS), and zymosan (ZYM), were diluted in PBS to a final concentration of 0.5 μg/μL. For PAMPs induction, 120 larvae on 3-day-old-fifth-instar were randomly divided into four groups. Each group was injected with 5 μL phosphate-buffered saline (PBS, negative control), LPS, PGN, or ZYM. Half an hour later, each group was reared on fresh mulberry leaves under standard conditions. At 12 h and 24 h post-injection, the fat bodies of each group were collected for RNA isolation.

### Cloning and Sequence Analysis

Based on the silkworm genome database (http://silkbase.ab.a.u-tokyo.ac.jp/cgi-bin/index.cgi), a pair of *KPI5*-specific primers was designed (**Table S1**). The cDNA obtained from the fat body of silkworms on 3-day-old-fifth-instar was used as the PCR template. PCR fragments were cloned into the pMD19-T vector (TaKaRa, Otsu, Japan) and sequenced. The signal peptides and functional domains of KPI5 were analyzed using the SMART program (http://smart.embl-heidelberg.de/). The theoretical isoelectric point (pI) and molecular mass of the mature KPI5 protein were predicted using the Expert Protein Analysis System (ExPASy, https://www.expasy.org/). Multi-sequence alignment analysis of KPI5 and other protease inhibitors from the Kunitz family was performed using ClustalX1.8.

### RNA Extraction, cDNA Synthesis, and Real-Time Quantitative PCR (RT-qPCR)

Total RNA of silkworm tissues was extracted using TRIzol reagent (Invitrogen, USA). cDNA was synthesized using TransScript One-Step gDNA Removal and cDNA Synthesis SuperMix (TransGen Biotech, Beijing, China) according to the manufacturer’s protocols. A qTOWER 2.2 REAL-TIME PCR Thermal Cycler System (Analytik Jena, Thuringia, Germany) and SYBR Premix Ex Taq Kit (TaKaRa, Shiga, Japan) were used for real-time PCR according to the manufacturer’s protocols. The optimal PCR amplification procedure consisted of an initial denaturation step of 30 s at 95°C and 40 amplification cycles consisting of 5 s at 95°C followed by 30 s at 60°C. The housekeeping gene *eIF4A* of *B. mori* was used as the internal reference gene (**Table S1**). Relative expression levels were calculated using the relative quantitative method (2^−ΔΔCt^).

### Recombinant Expression, Purification, Inhibitory Activity and Stability Analysis

The coding region of the *KPI5* gene without the signal sequence was amplified using a pair of *KPI5*-specific primers (**Table S1**). The amplified PCR product was cloned into the expression vector pET28a with restriction enzyme sites, *Nd*e I and *No*t I. The recombinant pET28a-*KPI5* plasmid was transformed into *E. coli BL21* (DE3) cells (Trans Gen Biotech, China) for protein expression. After induction with 1 mM isopropyl-β-D-thiogalactopyranoside (IPTG) at 37°C 4 h, the cell pellets were collected by centrifugation at 8000×g for 5 min. After sonication, the precipitated proteins were collected by centrifugation at 12000×g for 20 min. The precipitate was dissolved in 20 mL of renaturation buffer (8 M urea, 20 mM Tris-HCl, 100 mM NaCl, 2 mM reduced glutathione, 0.2mM oxidized glutathione). The dissolved supernatants were collected by centrifugation at 12000×g for 20 min and renatured by dialysis against renaturation buffer with decreasing concentrations of urea (6, 4, and 2 M). The final buffer consisted of 20 mM PBS (pH 7.4). Each dialysis step was performed at 4°C for 24 h. The refolded supernatant was filtered through a 0.45 μm microporous membrane, and the recombinant KPI5 (rKPI5) protein was preliminarily purified by Ni-NTA affinity chromatography. The crude rKPI5 protein was further purified using HiLoad 16/600 Superdex 75 pg chromatography (GE Healthcare, USA). The identity of the rKPI5 protein was confirmed by immunoblotting with an anti-histidine antibody. Rabbit polyclonal antibody against KPI5 was prepared by Zoonbio Biotechnology (Nanjing, China).

Protease inhibition assays were performed as previously described (35, 36), with slight modifications. rKPI5 protein (10 μg) was incubated with different commercial proteases (1 μg) in 100 μL assay buffer (100 mM Tris-HCl, pH 7.5) for 30 min at room temperature. A substrate of 10 ng FITC-casein (Thermo Fisher Scientific, USA) in 100 μL assay buffer was added, followed by incubation in the dark for 60 min at room temperature. Substrate hydrolysis was monitored using a microplate reader with excitation at 485 nm and emission at 535 nm. Protease inhibition by rKPI5 protein was assessed using the following formula: inhibition (%) = (1 – residual enzyme activity/enzyme activity without inhibitor)×100%.

### SDS-PAGE and Immunoblot Analysis

Silkworm tissue samples were lysed in RIPA lysis buffer (Beyotime, China), and protein concentrations were determined using a BCA assay kit (Beyotime Biotechnology, China). For SDS-PAGE, recombinant protein or tissue protein samples were treated with 5×SDS loading buffer for 5 min at 95°C and then separated by 15% (w/v) SDS-PAGE. Proteins were detected by staining with Coomassie brilliant blue. For immunoblot analysis, the proteins were transferred onto PVDF membranes. The PVDF membrane was blocked with 5% (w/v) skim milk powder in Tris-buffered saline containing 1% Tween 20 (TBST) for 2 h at 37°C. Then, the membranes were incubated with KPI5 antibody or gloverin 2 antibody (1:10,000) in TBST containing 1% (w/v) skim milk powder for 1-2 h at 37°C. The membranes were washed with TBST and incubated with HRP-labeled goat anti-rabbit IgG (1:20,000, Beyotime, China) for 1 h at 37°C. Signals were visualized with Super Signal West Femto Maximum Sensitivity Substrate (Thermo, USA) using a ChemiScope 3400 Mini instrument (Clinx Science, China).

### Microbial and PAMPs Binding Assay

*E. coli*, *S. aureus* and *S. cerevisiae* were cultured under appropriate conditions to obtain an OD of =0.6, and the pathogen pellet (1 mL culture medium) was collected by centrifugation at 6000×g for 5 min. The pellets were resuspended in 1 mL TBS and washed three times. Bacteria or fungus (500 μL) and rKPI5 protein (500 μL, 0.2 μg/μL) were incubated with rotation at 4°C for 2 h, and then centrifuged at 6000×g for 10 min. The pellet was washed with TBS three times, and the supernatants and pellets from the last washing were collected. The collected supernatant and pellet were boiled in SDS sample buffer for immunoblot analysis. Incubation with the same concentration and volume of pathogen and TBS buffer without rKPI5 protein was used as a negative control, and incubation with the same concentration and volume of rKPI5 protein was used as a positive control.

The binding between rKPI5 protein and PAMPs was measured using an enzyme-linked immunosorbent assay (ELISA) according to previous studies (37, 38), with slightly modifications. Briefly, 5 μg LPS, PGN, and ZYM were dissolved in coating solution (0.5 M carbonate buffer, pH 9.8), added to a 96-well microtiter plate, and incubated at 4°C overnight. Using PBST buffer (20 mM NaH_2_PO_4_, 20 mM Na_2_HPO_4_, and 0.05% Tween-20, pH 7.4) as washing solution, the microtiter plate was automatically washed five times in the ELx50 plate washer (BioTek Instruments, Inc., Winooski, VT) to remove uncoated PAMPs. Then, the coated PAMPs were incubated with blocking buffer (3% BSA and 10% normal goat serum in PBS) for 2 h at 37°C. After washing the microtiter plate five times with PBST, 100 μL of the rKPI5 protein in gradient dilution in PBS was added and incubated at 25°C for 2 h. After washing the microtiter plate five times with PBST, 1:1000 rabbit anti-KPI5 in PBS containing 1% BSA was added and incubated at 37°C for 1 h. After washing the microtiter plate three times with PBST, 100 μL of 1:4000 diluted HRP-labeled goat anti-rabbit IgG (Beyotime, China) was added and incubated at 37°C for 1 h. After washing the microtiter plate three times with PBST, 100 μL of TMB (3,3’,5,5’-tetramethylbenzidine) substrate solution was added and incubated in the dark at room temperature for 30 min. Finally, 50 μL 2 M H_2_SO_4_ was added to each well to stop the color reaction. Absorbance was measured at OD_450_ using a GloMax-Multi Detection System (Promega, Madison, WIa). Non-immune rabbit serum was used as a negative control and empty wells were used as a blank. Each experiment was performed in triplicate. Samples with (P_sample_ - B_blank_)/(N_negative_ - B_blank_) > 2.1 were considered positive (37).

### *In Vitro* Pathogen Growth Inhibition Assay and Encapsulation Analysis

*E. coli*, *S. aureus* and *S. cerevisiae* were cultured under appropriate conditions until they reached an OD of =0.3, and then were added to a 96-well microtiter plate together with the corresponding test sample, to a total volume of 200 μL. The negative control group was treated with 100 μL pathogen solution and 100 μL PBS, the positive control group was treated with 100 μL pathogen solution and 100 μL ethylenediaminetetraacetic acid (EDTA, 10 mM), and the experimental group was treated with 100 μL pathogen solution and 100 μL rKPI5 protein (final concentration 0.5 μg/μL). Each experiment was repeated in triplicate. Pathogen growth was observed using UV spectrophotometry by monitoring the absorbance at 600 nm (OD_600_). The bacterial culture group was cultured in a shaker at 37°C and 45 rpm, and the OD_600_ was measured every hour for a total of 6 h. The fungus group was cultured in a shaker at 28°C and 45 rpm, and the OD_600_ was measured every 12 h for a total of 72 h.

The *in vitro* encapsulation assay was performed as previously described (38, 39), with slight modifications. Briefly, Ni-NTA agarose beads (Qiagen, Dusseldorf, Germany) were equilibrated in TBS (Tris-HCl pH 8.0, 100mM NaCl), and rKPI5 protein was added and incubated with the beads at 4°C overnight. The recombinant protein-coated beads were washed three times with TBS and resuspended in TBS at a concentration of 80-100 beads/μL. A 48-well cell-culture plate was coated with 1% agarose. Hemolymph was collected from the fifth-instar larvae and mixed with an equal volume of Grace’s cell culture medium (10% FBS, 0.1% penicillin-streptomycin, and 200 μL 10 mM PTU). Hemocytes were allowed to adhere for at least 10 min at room temperature in each well. Subsequently, 1 μL of rKPI5-coated and rSPINK7-coated beads were added to each well, and the plate was incubated at room temperature. Beads without protein coatings were used as controls. Encapsulation of agarose beads was observed by microscopy at 6 h and 24 h after incubation. For each recombinant protein, the assay was performed in three wells.

### CRISPR/Cas9-Mediated Mutation

To knock out the *B. mori KPI5* gene using the CRISPR/Cas9 system, a specific target site was selected in the second exon region of *KPI5* gene using the CRISPR/Cas9 target online predictor (https://cctop.cos.uni-heidelberg.de:8043/). Based on the designed and synthesized single-guide RNA (sgRNA) fragment (**Table S1**), we constructed a *pBac*[3×P3-EGFP-U6-*KPI5* gRNA] plasmid that could synchronously express green fluorescent protein and *KPI5 gRNA* under the control of different promoters (40). The *pBac*[3×P3-EGFP-U6-*KPI5 gRNA*] and *piggyBac* helper plasmid (encoding piggyBac transposase) were mixed and microinjected into newly laid embryos (0-3 h post-oviposition) at a concentration of 400 ng/μL (41). The injected embryos (G0) were incubated at 27°C and 85% relative humidity for approximately 10 d until hatching. Hatched larvae were reared on fresh mulberry leaves under standard temperatures and photoperiods. G0 moths were sib mated or crossed with wild-type (WT) moths to obtain G1 embryos. G1 individuals that expressed green fluorescence in their eyes were screened using an Olympus SZX12 fluorescent stereomicroscope (Olympus, Japan). The G1 individuals that expressed *KPI5 gRNA* were bred and maintained. The gene-specific mutants for subsequent experiments were maintained by crossing the *KPI5 gRNA* transgenic strain with *nos-Cas9* transgenic strain (stored in our laboratory).

### Genotyping, Molecular Changes and Survival Analysis

Genomic DNA from WT and mutant larvae was extracted using Tissue DNA Kit (Omega Bio-tek, Norcross, GA). Genotyping of *KPI5* chimeric mutant individuals was performed by amplification with gene-specific primers which were designed at the sides of the gRNA site (**Table S1**). To confirm the mutation of the genome target, the PCR products were cloned into the pMD19-T vector (Takara, Shiga, Japan) and sequenced.

Individuals with different editing forms were dissected, and the hemolymph and fat body were collected on 3-day-old-fourth-instar. The expression of AMP genes (**Table S1**) was detected using RT-qPCR in control individuals and *KPI5* chimeric mutant individuals. Immunoblotting was used to detect the expression of KPI5 and gloverin 2 proteins in the hemolymph of control individuals and *KPI5* chimeric mutant individuals.

Survival analysis was performed to determine whether KPI5 affects the survival capability of silkworm larvae following a bacterial challenge. Thirty control individuals and *KPI5* chimeric mutant individuals on 3-day-old-fifth-instar were randomly selected. The larvae were injected with *S. aureus* (1×10^8^ CFU/mL, 10 μL) and larval survival was monitored every 12 h. The challenge test with *B. bombyseptieus* was performed by smearing mulberry leaves. 200 μL *B. bombyseptieus* was evenly spread on mulberry leaves of the same size. The mulberry leaves were fed to the larvae of each group, and the survival of larvae was monitored every 12 h.

### PO Activity Analysis

7.5 μg of rKPI5 protein was injected on 3-day-old-fifth-instar larvae, and the control group was injected with the same concentration and volume of BSA. Thirty minutes after injection, the hemolymph of silkworms in each group was collected and centrifuged at 4°C to remove hemocytes. Next, 5 μL cell-free hemolymph and 5 μL PAMPs (LPS, PGN, ZYM, 5 μL, 1 μg/μL) were successively added to a 96-well plate. Finally, 100 μL of 2 mM L-Dopa was added to determine its absorptivity at 490 nm within 30 min. PO activity was calculated as the change in absorbance per unit time. The method of determining PO activity in the hemolymph of wild-type silkworm and *KPI5* knockdown silkworm as descrived above.

## Results

### Cloning and Sequence Analysis of KPI5

**Figure 1:**
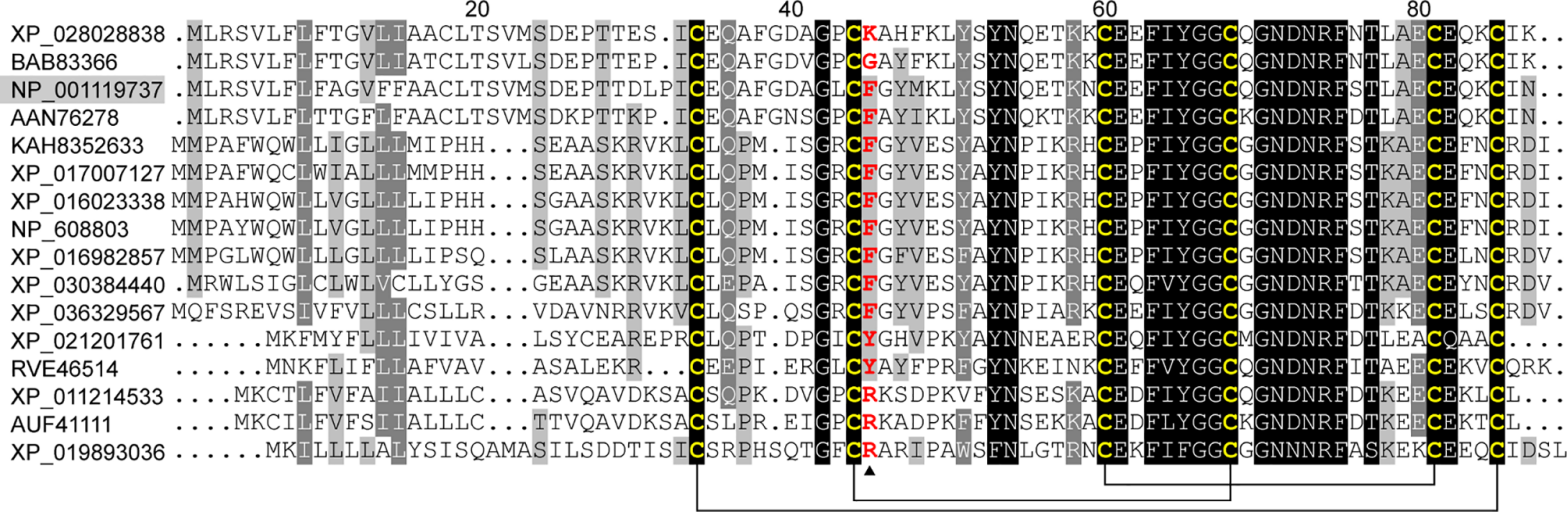
Sequence alignment of KPI5 with its homologs. Conserved, highly conserved and identical amino acid residues are highlighted in light gray, gray, and black, respectively. KPI5, *B. mori* (NP_001119737); SCI-I, *Bombyx mandarina* (XP_028028838); SCI-sb, *B. mori* (BAB83366); CI-b1, *B. mori* (AAN76278); SPI, *Musca domestica* (XP_019893036); KPI, *Drosophila pseudotakahashii* (KAH8352633); SCI-III, *Drosophila takahashii* (XP_017007127); KPI, *Helicoverpa armigera* (XP_021201761); SCI-III, *Drosophila simulans* (XP_016023338); SCI-III, *Scaptodrosophila lebanonensis* (XP_030384440); KPI, *Drosophila melanogaster* (NP_608803); SCI-II, *Rhagoletis pomonella* (XP_036329567); KPI, *Chilo suppressalis* (RVE46514); KPI, *Bactrocera dorsalis* (XP_011214533); KPI, *Anastrepha sororcula* (AUF41111); SCI-III, *Drosophila rhopaloa* (XP_016982857).The conserved cysteine residues and reactive sites are indicated with yellow and red (arrowhead), respectively. Disulfide linkages are indicated with solid lines.

*KPI5* contains a coding sequence of 261 bp that encodes an 86-amino-acid protein with a 23-amino-acid signal peptide. After removal of the N-terminal signal peptide, the mature protein of KPI5 was predicted to be 7117.84 Da with a theoretical pI of 4.09. Multiple sequence alignment showed that KPI5 shares similarities with the KPIs of *Musca domestica* (XP_019893036), *Anastrepha sororcula* (AUF41111), *Chilo suppressalis* (RVE46514), *Helicoverpa armigera* (XP_021201761), *Drosophila melanogaster* (NP_608803) and *Bombyx mandarina* (XP_028028838), with identities ranging from 39%-74% (**Figure 1**). These homologous KPIs possess six conserved cysteines that form three intramolecular disulfide bridges. A relative high sequence conservation was observed between the third and fifth cysteine residues in the Kunitz domain (**Figure 1**). The P1 site of KPI5 and several of its homologs have been predicted to be Phe^45^, suggesting that they may have inhibitory activity against chymotrypsin (42).

### Prokaryotic Expression, Inhibitory Activity and Stability Analysis of KPI5

**Figure 2:**
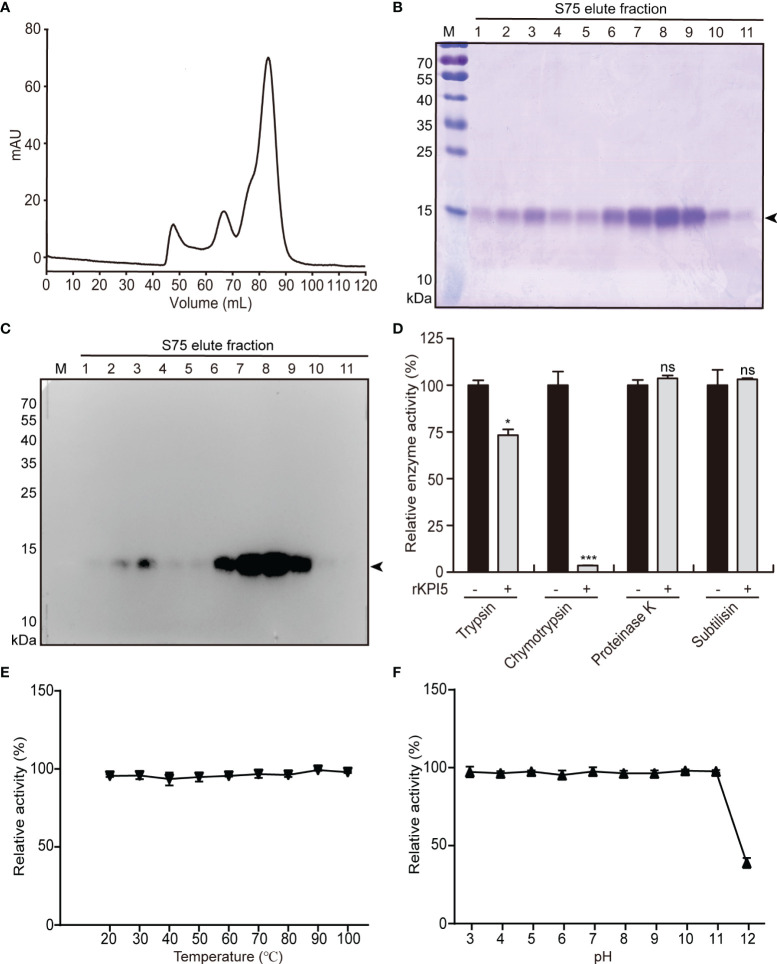
*Prokaryotic expression, inhibitory activity and stability analysis of KPI5.* **(A)** Purification of recombinant KPI5 (rKPI5) protein by gel filtration chromatography. **(B)** Coomassie brilliant blue staining and **(C)** immunoblot to detect purified rKPI5. **(D)** Inhibitory activity analysis of rKPI5 protein. Effects of temperature **(E)** and pH **(F)** on the activity of rKPI5 protein. The arrowhead indicates the rKPI5 protein. Significant differences are indicated as follows: *P < 0.05 and ***P < 0.001, and ns represents no significance.

*E. coli BL21 (DE3)* cells were used to express rKPI5. The rKPI5 in bacterial inclusion bodies was refolded by gradient dialysis and purified using Ni-NTA affinity chromatography. Crude-purified rKPI5 was collected and further purified by gel filtration (**Figure 2A**). After a two-step separation and purification, rKPI5 with high purity and specificity was obtained (**Figures 2B, C**).

To analyze the inhibitory activity of rKPI5 *in vitro*, FITC-casein was used as a substrate to test the inhibitory activity of rKPI5 against various commercial proteases. KPI5 showed strong inhibitory activity towards chymotrypsin and slight inhibitory activity towards trypsin, but not towards proteinase K and subtilisin (**Figure 2D**).

To evaluate the stability of KPI5 against changes in temperature and pH, commercial chymotrypsin was used in protease inhibition assays. First, rKPI5 was incubated at temperatures at 20-100°C for 10 minutes. Subsequently, the inhibitory activity against chymotrypsin was measured. The inhibitory activity of KPI5 remained unchanged with increasing temperatures, at all temperatures tested (**Figure 2E**). The effect of pH on the stability of KPI5 was investigated in a series of Britton-Robinson buffers with different pH values. The rKPI5 was incubated with a buffer of corresponding pH for 24 h at room temperature, and its inhibitory activity toward chymotrypsin was measured in PBS (20 mM, pH 7.4). KPI5 was very stable over a wide pH range (pH 3-11), although the inhibitory activity of KPI5 was significantly reduced to approximately 40% at pH 12 (**Figure 2F**).

### KPI5 Is Expressed Mainly in the Fat Body and Hemolymph During Larval Stage

**Figure 3:**
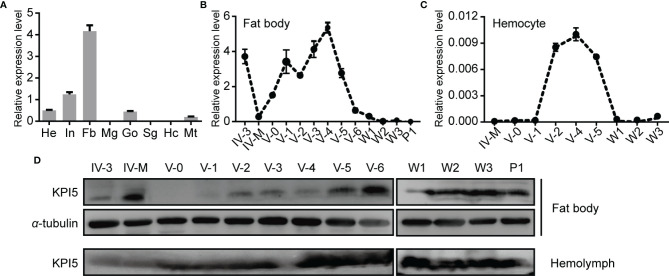
Expression profiles of KPI5 in different tissues and development stages of silkworm. **(A)** Expression profiles of KPI5 in various tissues from 3-day-old fifth-instar larvae. He, head; In, integument; Mg, midgut; Fb, fat body; Go, gonad; Sg, silk gland; Hc, hemocyte; Mt, Malpighian tubule. Temporal expression patterns analysis of *KPI5* in the fat body **(B)** and hemocyte **(C)** by real-time quantitative PCR. Immunoblot analysis of KPI5 protein in the fat body and hemolymph at different developmental stages **(D)**. IV-3, 3-day-old fourth-instar larvae; IV-M, molting fourth-instar larvae; V-0 to V-6, newly molted to 6-day-old fifth-instar larvae; W-1and W-3: days 1 and 3 after wandering; P-1: day 1 after pupation. *a*-tubulin was used as the reference protein.

The expression of KPI5 mRNA and protein in different tissues was analyzed using RT-qPCR and immunoblotting, respectively. The tissue expression profile showed that *KPI5* mRNA was mainly detected in the fat body, integument, head, and gonads of 3-day-old fifth-instar larvae (**Figure 3A**). Developmental expression profiles showed that *KPI5* was mainly expressed in the fat body of larvae during the feeding stage, whereas the expression level of *KPI5* in hemocytes during the fifth-instar stage was very low (**Figures 3B, C**). Furthermore, the protein levels of KPI5 in the fat body and hemolymph were detected by immunoblot analysis. The results showed that KPI5 protein could be detected continuously in the fat body and hemolymph from 3-day-old fourth-instar larvae to 1-day-old pupae (**Figure 3D**).

### KPI5 Binds to Pathogens and Their PAMPs, but Does Not Inhibit Pathogen Growth

**Figure 4:**
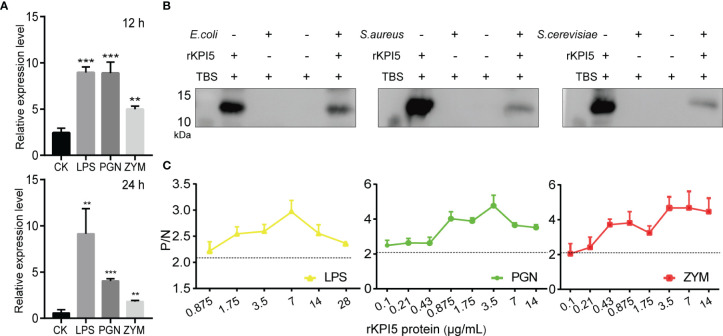
Binding analysis of recombinant KPI5 to pathogens and pathogen-associated molecular patterns (PAMPs). **(A)** Effect of PAMPs on the expression of *KPI5* in silkworm fat body. LPS, lipopolysaccharide, PGN, peptidoglycan, ZYM, zymosan. The control group was not injected with any PAMPs. Significant differences are indicated as follows: **P < 0.01 and ***P < 0.001. **(B)** Immunoblot analysis of the interaction between recombinant KPI5 and bacteria. **(C)** Enzyme-linked immunosorbent assay **(**ELISA) analysis of the interaction between rKPI5 and bacteria PAMPs. Pre-immune serum protein was used as the negative control group, and diluent PBST was used as the blank control group. Samples with (P_sample_ - B_blank_)/(N_negative_ - B_blank_) > 2.1 were considered positive.

To examine whether KPI5 plays a role in immune defense in the fat body of silkworms, the expression level of *KPI5* after PAMP challenge was determined using RT-qPCR. Compared to the control, *KPI5* mRNA levels were significantly upregulated at 12 and 24 h after injection with LPS, PGN, and ZYM (**Figure 4A**).

To test whether the KPI5 protein functions by directly binding to pathogens and their PAMPs, immunoblot and enzyme-linked immunosorbent assay (ELISA) were used to analyze the interaction between KPI5 and pathogens and their PAMPs. Immunoblot analysis showed that KPI5 exhibited positive binding to *E. coli*, *S. aureus*, and *S. cerevisiae* (**Figure 4B**). The ELISA results showed that KPI5 exhibited positive binding with LPS, PGN, and ZYM at different concentrations (0.875-28 μg/mL) (**Figure 4C**). These results showed that KPI5 directly binds to pathogens and their PAMPs.

To detect whether KPI5 has antimicrobial activity after binding to pathogens, *E. coli*, *S. aureus*, and *S. cerevisiae* were incubated with 0.5 μg/μL KPI5. The results showed that KPI5 had no significant inhibitory effect on *E. coli*, *S. aureus*, and *S. cerevisiae* growth (**Figure S1A**). Furthermore, a hemocyte encapsulation experiment was performed to analyze whether KPI5 mediates cellular immunity after binding to pathogens. The results showed that KPI5 did not promote hemocyte encapsulation or melanization (**Figure S1B**). These results suggest that KPI5 neither participates in cellular immunity nor acts as an immune effector molecule to kill pathogens.

### CRISPR/Cas9-Mediated* KPI5 *Knockout

**Figure 5:**
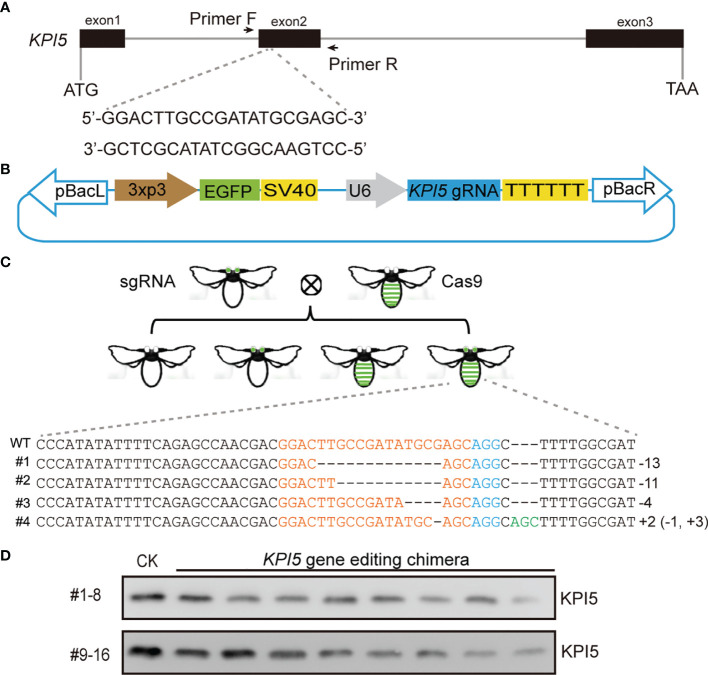
CRISPR/Cas9-mediated *KPI5* knockout. Schematic diagram of *KPI5*-gRNA location **(A)** and pBac-based *KPI5-*gRNA expressing vector construction **(B)**. EGFP was used as a selection marker. *KPI5-gRNA* expression cassettes were driven by a U6 promoter. Primer F and Primer R were used to detect the edited form of *KPI5* gene. **(C)** Edited form of *KPI5* gene in F1 generation chimeric mutant individuals. **(D)** Detection of KPI5 protein in control individuals and F1 generation chimeric mutant individuals.

To explore the physiological functions of silkworm KPI5, we performed CRISPR/Cas9-meidated genome editing to knock out KPI5. First, a gRNA was designed against the second exon of the *KPI5* gene, and a pBac-based *KPI5-gRNA* expressing vector was constructed (**Figures 5A, B**). Second, the vector was injected into *B. mori* G0 eggs, and G1 individuals with sable green fluorescence in the eyes were hybridized with a *nos-Cas9* transgenic strain exhibiting sable green fluorescence in the integument to produce F1 individuals (**Figure 5C**). Third, the genomic DNA of F1 individuals with stable green fluorescence in the eyes and integument were extracted from exuviae for PCR amplification of the *KPI5* gene, and the chimeric mutant individuals were screened by PAGE and direct sequencing analysis of PCR products (**Figure 5C**). Finally, the hemolymph of the chimeric mutant individuals was collected to detect the expression of KPI5 protein in 3-day-old fourth-instar larvae (**Figure 5D**). The results showed that KPI5 protein was significantly reduced in the hemolymph of chimeric mutant individuals compared to that in control individuals (**Figure 5D**).

### KPI5 Promotes the Expression of AMPs in the Silkworm Fat Body

**Figure 6:**
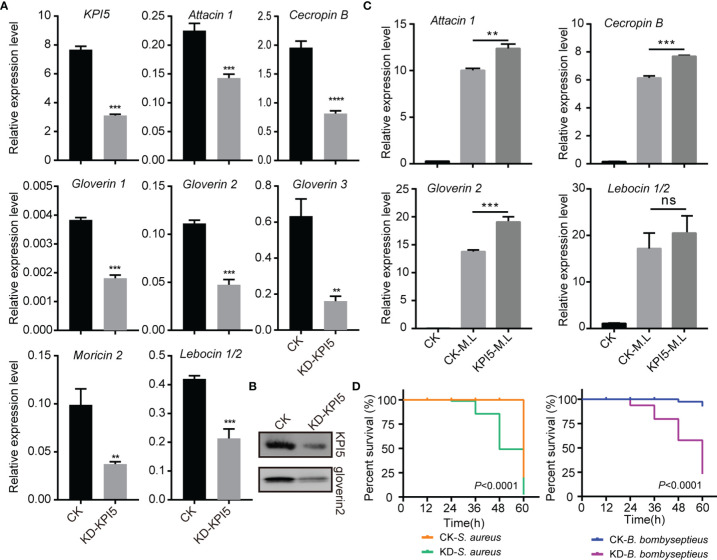
Effects of *KPI5* knockdown on antimicrobial peptide (AMP) expression and resistance of silkworm to pathogen. **(A)** RT-qPCR analysis of AMP expression in fat body after *KPI5* knockdown. Significant differences are indicated as follows: **P < 0.01, ***P < 0.001 and ****P < 0.0001, and ns represents no significance. **(B)** Immunoblot analysis of KPI5 and gloverin 2 protein in hemolymph after *KPI5* knockdown. **(C)** Effect of pre-injection of KPI5 protein into *B. mori* on the expression of AMPs induced by heat-inactivated *M. luteus*. **(D)** After knockdown of KPI5, *S. aureus* or *B*. *bombyseptieus* was administered, and the survival curves were counted. Statistical analysis between experimental (KD) and control (CK) groups were calculated by the log-rank test (Mantel-Cox, n = 30).

To analyze the effect of KPI5 on humoral immunity, we first examined the expression levels of AMPs in *KPI5* chimeric mutant individuals. The results revealed that the expression of AMP genes, including *attacin 1*, *cecropin B*, *gloverin 1*, *gloverin 2*, *gloverin 3*, *lebocin1/2*, and *moricin 2*, was remarkably downregulated in the fat body of *KPI5* chimeric mutant individuals compared to the control individuals (**Figure 6A**). Immunoblot analysis showed that KPI5 protein and gloverin 2 proteins were significantly reduced in the hemolymph of *KPI5* chimeric mutant individuals compared to control individuals (**Figure 6B**). To further analyze the regulation of KPI5 on the expression of AMPs, we detected the effect of pre-injection of rKPI5 protein into *B. mori* on the expression of AMPs induced by heat-inactivated *M. luteus* (an immune response inducer). The results showed that the expression of *attacin 1*, *cecropin B*, and *gloverin 2* was significantly higher in the pre-injected rKPI5 group than in the control group (**Figure 6C**). These results suggest that KPI5 promotes AMP gene expression in the silkworm fat body.

Furthermore, control individuals and *KPI5* chimeric mutant individuals were challenged with *S. aureus* or *B. bombyseptieus* (a bacterial pathogen of the silkworm), and the survival rate was determined. The results revealed that *KPI5* chimeric mutant group’s survival rate treated with *S. aureus* or *B. bombyseptieus* was significantly lower than that of the control group (**Figure 6D**).

### KPI5 Inhibits PO Activity and Melanization in Silkworm Hemolymph

**Figure 7:**
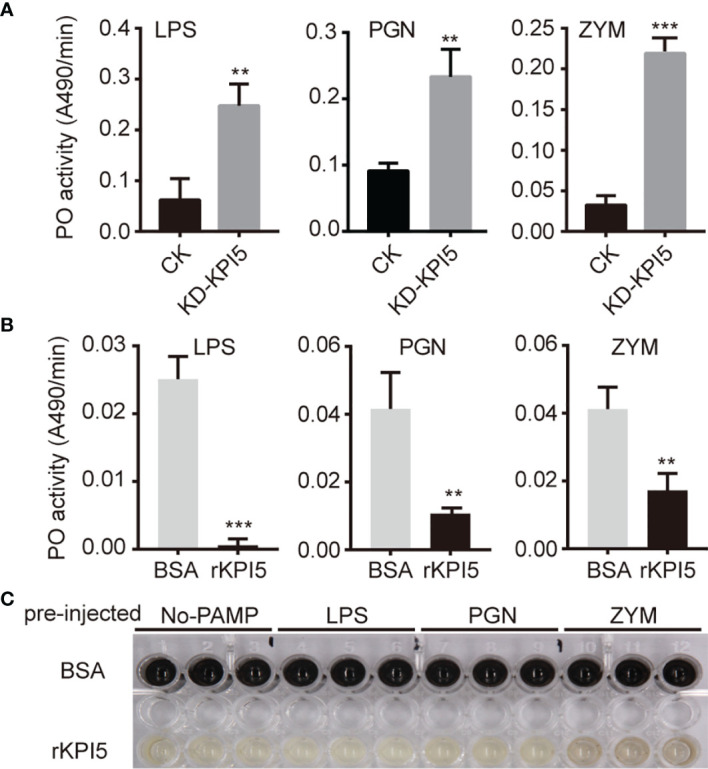
Effects of KPI5 on PO activity and melanization in hemolymph of silkworm. **(A)** Effect of *KPI5* knockdown on PO activity in hemolymph of silkworm. Effects of pre-injection of KPI5 protein into *B. mori* on PO activity **(B)** and melanization **(C)** in hemolymph induced by pathogen-associated molecular patterns. LPS, lipopolysaccharide; PGN, peptidoglycan; ZYM, zymosan. L-dopa was used as a substrate for the determination of PO activity. PO activity was assayed on a plate reader and shown as mean ± SD (n = 3) in the bar graphs. Significant differences are indicated as follows: **P < 0.01 and ***P < 0.001.

Previous studies have shown that insect endogenous KPIs may be involved in the regulation of the activation of proPO (24, 25). Therefore, we examined the hemolymph PO activity in control individuals and *KPI5* chimeric mutant individuals. The results showed that PO activity in the hemolymph of *KPI5* chimeric mutant individuals was significantly higher than that of control individuals after LPS, PGN, and ZYM induction (**Figure 7A**).

To further analyze the regulation by KPI5 of PO activity in the hemolymph, we detected the effect of pre-injection of KPI5 protein into *B. mori* on PO activity induced by LPS, PGN, and ZYM. The results showed that hemolymph PO activity in the pre-injected KPI5 group was significantly lower than that in the control group, and pre-injected KPI5 significantly inhibited hemolymph melanization (**Figures 7B, C**).

## Discussion

KPIs are an important group of ubiquitous protease inhibitors that are found in microbes to mammals. In invertebrates, KPIs, as well as having protease inhibitor activity, are involved in a range of diverse functional roles (29). Previous studies have shown that KPIs are a class of protease inhibitors that exist in the hemolymph of Lepidoptera and Diptera species (24, 43–46). However, it is unclear whether these proteins function in insect immune defense. In this study, we focused on the hemolymph protein KPI5 in silkworms, and explored its function in innate immunity.

The Kunitz domain is a cysteine-rich peptide chain of  approximately  60 amino acid residues and is stabilized by three conserved disulfide bridges (27). Multiple sequence alignment revealed that KPI5 and its homologs have typical Kunitz sequence characteristics, namely six conserved cysteines. The formation of multiple pairs of disulfide bonds by conserved cysteine residues is a feature shared by several low molecular weight protease inhibitor families, including Kazal, TIL, FPI, and Kunitz (15). Like FPI, TIL and other Kunitz molecules (35, 47), KPI5 displays high thermal and pH stability, which may be attributable to multiple disulfide bonds. The nature of amino acids at the P1 site determines the inhibition specificity of KPIs. For KPIs, when the P1 site is arginine or lysine, it can inhibit the activity of trypsin; when the P1 site is histidine, aspartic acid, leucine, phenylalanine or tryptophan, it can inhibit the activity of chymotrypsin (26, 29, 42). KPI5 exerts strong inhibitory activity against chymotrypsin, which is determined by the phenylalanine residue at the P1 site. In terms of molecular mechanism of action, KPIs participate in various physiological processes mainly by inhibiting the activity of serine proteases (trypsin, chymotrypsin, elastase, and kallikrein) (29). In addition, some KPIs are involved in regulating kinase signal transduction and blocking ion channels by unknown mechanisms (27, 28). This indicates that KPIs may have diverse mechanisms of action.

Our results showed that KPI5 was significantly upregulated by LPS, PGN, and ZYM, suggesting that it is involved in the immune response of the silkworm. We further found that KPI5 binds to pathogens and their PAMPs but does not inhibit pathogen growth. Unlike KPI5, silkworm low molecular weight protease inhibitors SPINK7 (Kazal-type) and BmSPI51 (Kunitz-type) directly inhibit fungal growth by binding to *β*-d-glucan on the fungal surface (30, 38). Insect immune processes mainly include: immune recognition, signal transmission, and effector production (48). Previous studies have shown that pattern recognition proteins (PRPs) in insects activate immune pathways by binding to PAMPs (49). BmPGRP-S1 can be used as a pattern recognition protein to bind bacteria or PG and activate the IMD pathway to activate the production of AMPs (50). Our results showed that KPI5 can promoted AMP expression in the silkworm fat body. Based on these results, we found that the functional mode of KPI5 is similar to that of PGRPs, that is, they first recognize pathogens and then promote the expression of effector molecules. Therefore, we speculated that KPI5 may be a new PRP in the immune response of silkworms. However, the molecular mechanism by which KPI5 regulates the expression of antimicrobial peptides still needs further study.

In the insect serine protease cascade, non-self-recognition often activates the production of antimicrobial peptides and melanization. Previous studies have shown that KPI5 exists in the active ingredient of PPO activation (25). Our results indicated that KPI5 inhibits hemolymph PO activity and melanization in silkworms. When the melanization reaction is overactivated, more cytotoxic substances are produced. Excess reactive oxygen species and quinine cause damage to insect tissue functions. Therefore, melanization reaction of insect hemolymph must be strictly regulated. Previous studies have shown that several serpins are involved in regulating PPO activation by inhibiting EPCs (15, 19, 36, 51–54). The expression of these serpins is typically upregulated by immune challenges, indicating a pathogen-induced negative feedback inhibitory mechanism (55). Our results directly confirm that insect KPIs are also involved in the regulation of hemolymph melanization. Like inducible serpins, the expression of KPI5 was also upregulated by immune challenges, but its protein abundance was significantly higher in normal silkworm larvae hemolymph than that of serpins (56). This suggests that KPI5 may play a more important role in avoiding excessive melanization of silkworm hemolymph. Based on the well-established PPO activation pathway of *M. sexta*, hemolymph protease 14 (HP14) senses non-self-presence and triggers a branched serine protease activation pathway, which leads to PPO activation and melanin formation (14, 57). *M. sexta* HP14 contains five low-density lipoprotein receptor class A repeats, a sushi domain, a unique Cys-rich region, and a typical chymotrypsin-like serine protease domain (57, 58). *M. sexta* HP14 is the only chymotrypsin in the currently reported insect PPO activation pathway, and *B. mori* SP14 is highly similar to HP14 (57–59). Therefore, we speculated that KPI5, a chymotrypsin inhibitor, might be involved in the regulation of melanization by inhibiting the activity of SP14 in silkworms.

Overall, we found that KPI5 is synthesized in the fat body and secreted into the hemolymph. KPI5 was strongly upregulated in the fat body after PAMPs challenge. Additionally, KPI5 was able to bind to PAMPs, but did not inhibit pathogen growth. Finally, KPI5 plays a dual regulatory role in pathogen invasion by promoting the expression of antimicrobial peptides in the fat body and inhibiting hemolymph melanization. Our results show that KPI5 has two contrasting regulatory functions in the silkworm immune response. We speculated on the possible regulatory mechanism of KPI5: on one hand, it inhibits the activation of hemolymph PPO, regulates the level of melanization reaction, and avoids excessive melanization; on the other hand, it promotes the production of antimicrobial peptides to kill pathogens and restores the immune homeostasis of the silkworm. However, the molecular mechanism by which KPI5 exerts its dual regulatory roles remains unclear. Further investigation of the interacting proteins or physiological target proteases of KPI5 will contribute to a better understanding of the molecular mechanisms underlying insect immune regulation.

## Data Availability Statement

The original contributions presented in the study are included in the article/**Supplementary Material**. Further inquiries can be directed to the corresponding author.

## Author Contributions

HL, QX, and PZ contributed to conception and design of the study. JH and HL wrote the manuscript. JH, HL, JX, XH, XS, and RY performed the research and analyzed the data. All authors contributed to the article and approved the submitted version.

## Funding

The authors would like to thank grants from the National Natural Science Foundation of China (32030103; 32172798), the Fundamental Research Funds for the Central Universities (SWU-KQ22008), the Chongqing Science and Technology Commission (cstc2020jcyj-cxttX0001) and National Training Program of Innovation and Entrepreneurship for Undergraduates (202010635030).

## Conflict of Interest

The authors declare that the research was conducted in the absence of any commercial or financial relationships that could be construed as a potential conflict of interest.

## Publisher’s Note

All claims expressed in this article are solely those of the authors and do not necessarily represent those of their affiliated organizations, or those of the publisher, the editors and the reviewers. Any product that may be evaluated in this article, or claim that may be made by its manufacturer, is not guaranteed or endorsed by the publisher.

## Supplementary Material

Supplementary Figure 1 Antimicrobial activity and encapsulation analysis of recombinant KPI5 (rKPI5). **(A)** The inhibitory effect of rKPI5 on the growth of *E. coli*, *S. aureus*, and *S. cerevisiae*. **(B)** Encapsulation of rKPI5 or rSPINK7-coated beads by hemocytes. SPINK7 was used as a positive control. SPINK7-coated beads were encapsulated and melanized by *B. mori* hemocytes. KPI5-coated beads were not encapsulated and melanized by *B. mori* hemocytes.

Supplementary Table 1 Primers used in this study.
