# Supplementary figures and images for: KPI5 Is Involved in the Regulation of the Expression of Antibacterial Peptide Genes and Hemolymph Melanization in the Silkworm, Bombyx mori
Source: Front Immunol. 2022 May 20;13:907427. doi: 10.3389/fimmu.2022.907427 (PMC9164257; doi:10.3389/fimmu.2022.907427)

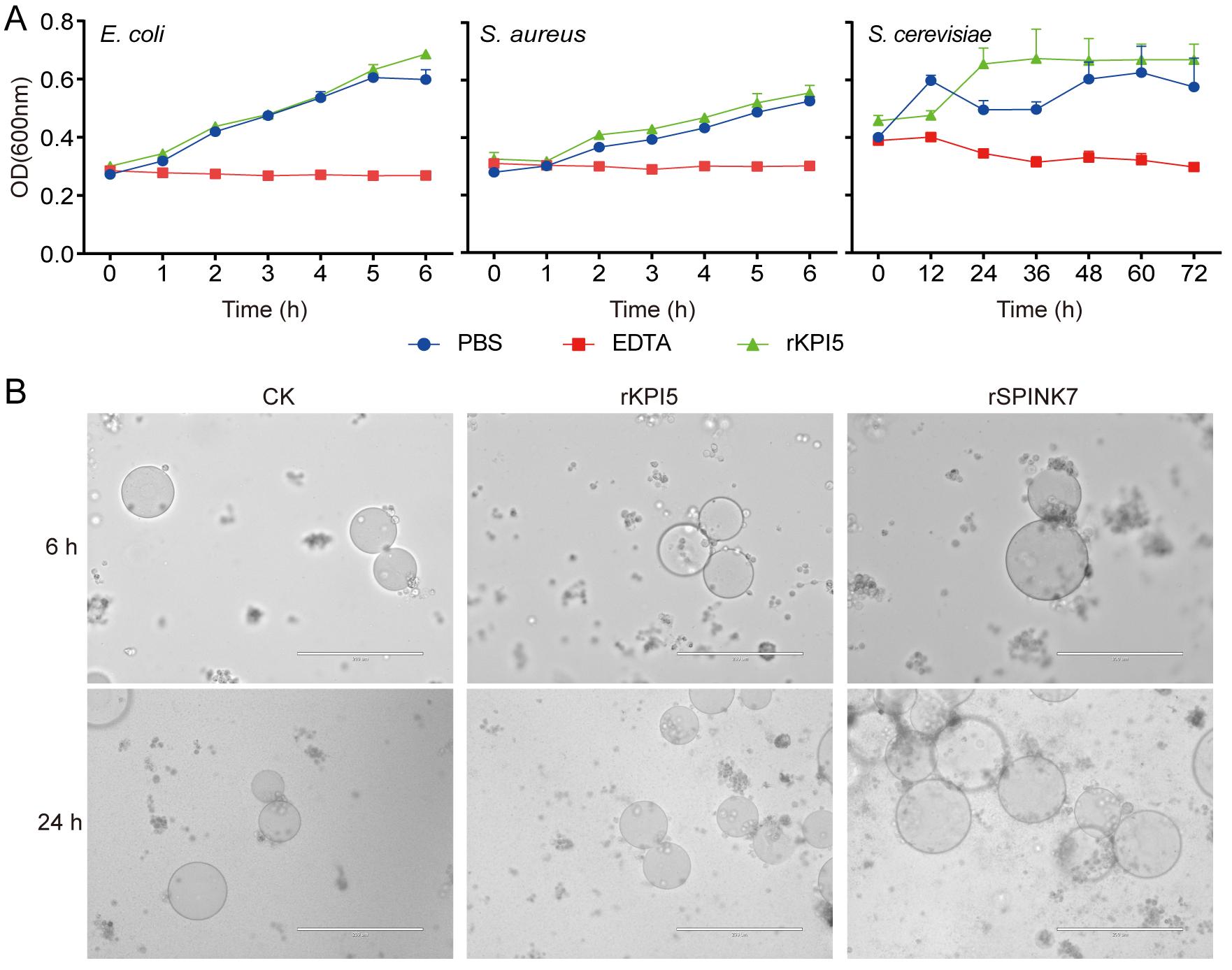

Supplement: Supplementary Figure 1 — Antimicrobial activity and encapsulation analysis of recombinant KPI5 (rKPI5). (A) The inhibitory effect of rKPI5 on the growth of E. coli, S. aureus, and S. cerevisiae. (B) Encapsulation of rKPI5 or rSPINK7-coated beads by hemocytes. SPINK7 was used as a positive control. SPINK7-coated beads were encapsulated and melanized by B. mori hemocytes. KPI5-coated beads were not encapsulated and melanized by B. mori hemocytes. [file Image_1.tif]
