# Supplementary material for: KPI5 Is Involved in the Regulation of the Expression of Antibacterial Peptide Genes and Hemolymph Melanization in the Silkworm, Bombyx mori
Source: Front Immunol. 2022 May 20;13:907427. doi: 10.3389/fimmu.2022.907427 (PMC9164257; doi:10.3389/fimmu.2022.907427)
Supplement: Supplementary Table 1 — Primers used in this study. [file Table_1.docx]

**Table S1 Primers used in this study**

| Primer name | Purpose | Direction | Primer sequence (5’−3’) | Remarks |
| --- | --- | --- | --- | --- |
| KPI5 | cloning | Forward | ATGTTGAGATCAGTGCTTTTT |  |
|  |  | Reverse | TTAGTTTATGCATTTCTGTTCGC |  |
| rKPI5 | prokaryotic expression | Forward | CGGGATCCCATATGGATGAGCCAACGACGGACTT | *Nde* I |
|  |  | Reverse | GCTCTAGAGCGGCCGCTTAGTTTATGCATTTCTGTTCGC | *Not* I |
| KPI5 | gRNA vector | Forward | AAGTGGGACTTGCCGATATGCGAGC |  |
|  |  | Reverse | AAACGCTCGCATATCGGCAAGTCCC |  |
| KPI5 | Detecting forms of gene editing | Forward | CAAGTGGATTCCGGTGAC |  |
|  |  | Reverse | CATTCGGGTTGTCGTGTT |  |
| BmeIF4A | RT-qPCR (reference gene) | Forward | TTCGTACTGGCTCTTCTCGT |  |
|  |  | Reverse | CAAAGTTGATAGCAATTCCCT |  |
| KPI5 | RT-qPCR | Forward | GCGATGCGGGTCTTTGTT |  |
|  |  | Reverse | ACCAGGGCAGCCGATTTA |  |
| Attacin 1 |  | Forward | CTGAGCCTGACCGGGAC |  |
|  |  | Reverse | AGGTCGTGGTTGTTATTGTG |  |
| Cecropin B |  | Forward | CTATCCTTCGTCTTCGCTCT |  |
|  |  | Reverse | ATAGCTTTAGCCGAACCGAG |  |
| Gloverin 1 |  | Forward | AGCCAATTTTCAAAGTCAGTCC |  |
|  |  | Reverse | TCATTGAAGAACTCCCTGTTGT |  |
| Gloverin 2 |  | Forward | TGTGTTAACGCAGAAGTTTACG |  |
|  |  | Reverse | GTAACCAGCTTTTCCAAAGAGG |  |
| Gloverin 3 |  | Forward | AAGCTGCCATTGACATAAACAG |  |
|  |  | Reverse | GGGTGTTCTTATCAAGATCCCA |  |
| Moricin 2 |  | Forward | TGTGGCAATGTCTCTGGTGT |  |
|  |  | Reverse | GCTTTCTTTTCTTCGGTTTCAA |  |
| Lebocin 1/2 |  | Forward | CGTTTAACCCCAAGCCAATA |  |
|  |  | Reverse | TGCACTCCGAAATCTTTTGT |  |
